# Southern African HIV Clinicians Society guideline for the prevention, diagnosis and management of cryptococcal disease among HIV-infected persons: 2019 update

**DOI:** 10.4102/sajhivmed.v20i1.1030

**Published:** 2019-11-08

**Authors:** Nelesh P. Govender, Graeme Meintjes, Phetho Mangena, Jeremy Nel, Samantha Potgieter, Denasha Reddy, Helena Rabie, Douglas Wilson, John Black, David Boulware, Tom Boyles, Tom Chiller, Halima Dawood, Sipho Dlamini, Thomas S. Harrison, Prudence Ive, Joseph Jarvis, Alan Karstaedt, Matamela C. Madua, Colin Menezes, Mahomed-Yunus S. Moosa, Zaaheera Motlekar, Amir Shroufi, Sarah Lynn Stacey, Merika Tsitsi, Gilles van Cutsem, Ebrahim Variava, Michelle Venter, Rachel Wake

**Affiliations:** 1 National Institute for Communicable Diseases, a Division of the National Health Laboratory Service, Johannesburg, South Africa; 2 School of Pathology, University of the Witwatersrand, Johannesburg, South Africa; 3 Division of Medical Microbiology, University of Cape Town, Cape Town, South Africa; 4 Institute of Infectious Disease and Molecular Medicine, University of Cape Town, Cape Town, South Africa; 5 Department of Medicine, University of Cape Town, Cape Town, South Africa; 6 Department of Medicine, Polokwane Hospital, Polokwane, South Africa; 7 Helen Joseph Hospital, University of the Witwatersrand, Johannesburg, South Africa; 8 Department of Internal Medicine, University of the Free State, Bloemfontein, South Africa; 9 Division of Infectious Diseases, Department of Internal Medicine, Chris Hani Baragwanath Academic Hospital, University of the Witwatersrand, Johannesburg, South Africa; 10 Department of Paediatrics, Tygerberg Hospital, Stellenbosch University, Stellenbosch, South Africa; 11 Department of Internal Medicine, Edendale Hospital, Pietermaritzburg, South Africa; 12 School of Clinical Medicine, University of KwaZulu-Natal, Pietermaritzburg, South Africa; 13 Department of Infectious Diseases, Livingstone Hospital, Port Elizabeth, South Africa; 14 Department of Medicine, Centre for Infectious Diseases and Microbiology Translational Research, University of Minnesota, Minneapolis, United States; 15 Anova Health Institute, Johannesburg, South Africa; 16 Faculty of Health Sciences, University of the Witwatersrand, Johannesburg, South Africa; 17 Mycotic Diseases Branch, US Centres for Disease Control and Prevention, Atlanta, United States; 18 Department of Medicine, Grey’s Hospital, Pietermaritzburg, South Africa; 19 Caprisa, University of KwaZulu-Natal, Pietermaritzburg, South Africa; 20 Division of Infectious Diseases and HIV Medicine, Department of Medicine, Groote Schuur Hospital, University of Cape Town, Cape Town, South Africa; 21 Centre for Global Health, Institute of Infection and Immunity, St George’s University of London, London, United Kingdom; 22 Division of Infectious Diseases, Department of Internal Medicine, School of Clinical Medicine, Faculty of Health Sciences, Helen Joseph Hospital, Johannesburg, South Africa; 23 Department of Clinical Research, Faculty of Infectious and Tropical Diseases, London School of Hygiene and Tropical Medicine, London, United Kingdom; 24 Division of Infectious Diseases, Department of Internal Medicine, Charlotte Maxeke Johannesburg Hospital, Johannesburg, South Africa; 25 Department of Medicine, Rob Ferreira Hospital, Mbombela, South Africa; 26 Department of Infectious Diseases, Nelson R. Mandela School of Medicine, University of KwaZulu-Natal, Durban, South Africa; 27 Department of Medicine, Kimberley Provincial Hospital, Kimberley, South Africa; 28 Department of Internal Medicine, Charlotte Maxeke Johannesburg Academic Hospital, Johannesburg, South Africa; 29 Southern Africa Medical Unit, Médecins Sans Frontières, Cape Town, South Africa; 30 Centre for Infectious Disease Epidemiology and Research, University of Cape Town, Cape Town, South Africa; 31 Department of Medicine, Tshepong Hospital, Klerksdorp, South Africa

## Introduction

### Rationale for guideline update

Six years after the Southern African HIV Clinicians Society cryptococcal disease guideline was published in 2013, cryptococcal meningitis (CM) remains an important cause of mortality among antiretroviral treatment (ART)-naïve and ART-experienced HIV-seropositive adults in South Africa.^1,2^ Several important practice-changing developments led us to update the guideline to diagnose, prevent and manage this common fungal opportunistic infection. The World Health Organization (WHO) published a guideline for advanced HIV disease in 2017 and a guideline relevant to resource-limited settings for HIV-associated CM in 2018.^3,4^ Cryptococcal antigen (CrAg) screening and pre-emptive treatment reduced all-cause mortality among ambulatory participants in a randomised clinical trial in Zambia and Uganda.^5^ Following an evaluation of reflex versus provider-initiated screening, national reflex laboratory CrAg screening was implemented in South Africa in 2016.^6,7^ Recently completed clinical trials conducted in resource-limited settings have provided evidence for the best first-line antifungal regimens for CM and the role of corticosteroids in CM.^8,9^ Finally, international and local advocacy efforts have resulted in increasing, yet still limited, access to flucytosine and a reduced cost of liposomal amphotericin B for the treatment of CM.^10^

### Summary of major changes to the guideline

1. We now recommend CrAg screening for all adults or adolescents with a CD4_+_ T-lymphocyte (CD4) count < 200 cells/µL who are initiating ART for the first time, switching after ART failure or re-entering into care after prior disengagement.
2. Reflex laboratory CrAg screening is recommended as the preferred approach in South Africa, although alternative approaches may be more suitable for other countries in Southern Africa.
3. To diagnose CM, lumbar puncture (LP) is recommended for all patients with a new positive CrAg screening test result.
4. The recommended induction regimen for CM is 1 week of amphotericin B deoxycholate (1 mg/kg/day) and flucytosine (100 mg/kg/day in four divided doses), followed by 1 week of fluconazole (1200 mg daily for adults; 12 mg/kg/day for children and adolescents up to a maximum of 800 mg daily).
5. We have recommended alternative induction regimens for CM among patients with renal dysfunction, including liposomal amphotericin B-based options.
6. A higher dose of fluconazole is now recommended during the 8-week consolidation phase for CM (800 mg daily for adults; 12 mg/kg/day for children and adolescents up to a maximum of 800 mg daily).
7. Maintenance treatment for CM is recommended to be continued for at least 12 months and until a single CD4 count is > 200 cells/µL and the HIV viral load is suppressed.
8. We have included new recommendations for the prevention, monitoring and management of flucytosine toxicities.
9. Although a manometer is the most accurate way to measure raised intracranial pressure, we have suggested alternative options for assessment of elevated pressure when it is unavailable.
10. Patients with a positive blood CrAg test result in whom CM is ruled out by LP (a negative cerebrospinal fluid [CSF] CrAg test is the most rapid method to establish this) should be given oral fluconazole alone as induction therapy (adults: 1200 mg for 2 weeks). Antiretroviral treatment may be commenced immediately.
11. We recommend immediate referral for LP in all patients with a new positive blood CrAg test who initiated ART within the 4-week period prior to the CrAg test. Among those with a negative CSF CrAg test (i.e. in whom CM is excluded), ART is recommended to be continued and fluconazole pre-emptive therapy should be initiated. Among those with a new diagnosis of CM during the first 4 weeks of ART, the guideline panel thought that there was clinical equipoise in terms of a decision to continue or interrupt ART.
12. Antifungal susceptibility testing is now recommended if a patient has a single relapse episode of CM and other causes have been excluded.

## Recommendation 1: Cryptococcal antigen screening and pre-emptive treatment

### Background

Early diagnosis of HIV infection and early initiation of ART before immunosuppression is the most important strategy to reduce the incidence of CM and CM-associated mortality. In South Africa, patients should initiate ART according to the current national guideline.^1,11,12^ Screening for subclinical cryptococcal disease has a proven mortality benefit among HIV-seropositive patients with low CD4 counts. In the Reduction of Early Mortality among HIV-infected Subjects sTarting AntiRetroviral Therapy (REMSTART) trial, HIV-seropositive outpatients with a CD4 count < 200 cells/µL, who were randomised to receive CrAg screening and community-based ART adherence support, had a 28% reduced all-cause mortality rate compared to those receiving standard of care (intervention: 134/1001 [13%] vs. standard: 180/998 [18%]).^5^ While the previous Southern African guideline recommended CrAg screening for patients with CD4 < 100 cells/µL, this approach will fail to detect a substantial proportion of cases that occur in patients with CD4 counts between 100 cells/µL and 200 cells/µL. A meta-analysis estimated that the pooled global prevalence of cryptococcal antigenaemia was 6.5% among those with CD4 count < 100 cells/µL and 2% among those with a CD4 count between 101 cells/µL and 200 cells/µL.^13^ A post hoc analysis of REMSTART trial data revealed an important mortality benefit of CrAg screening and pre-emptive treatment in subgroups of patients with a CD4 count < 100 cells/µL and with CD4 count of 101 cells/µL – 200 cells/µL.^4^ Thus, we now recommend that 200 cells/µL should be used as the CD4 threshold below which patients should be screened for cryptococcal disease, where resources permit (Table 1).

**TABLE 1:** Summary of recommendation 1.

Scenario	Sub-recommendations
HIV-seropositive adults or adolescents (≥ 10 years) with a CD4 count < 200 cells/µL	Screen for cryptococcal antigenaemia on serum or plasma by reflex laboratory testing (preferred) or clinician-initiated testing If clinician-initiated testing is performed, screening should be restricted to adults or adolescents without prior cryptococcal disease who are initiating or re-initiating ART A cryptococcal antigen lateral flow assay is the preferred method for screening (vs. a latex agglutination test format)
HIV-seropositive children (< 10 years)	There are insufficient data to recommend routine cryptococcal antigen screening in children
Patients with a new *positive* CrAg test result	Refer to Figure 1 and Recommendations 1, 3 and 5 regarding further investigations, antifungal treatment and timing of ART
ART was started before a new CrAg-positive result was received	Immediately refer for LP all patients with a positive blood CrAg who initiated a new ART regimen in the previous 4 weeks
Patients with a negative CrAg test result	Evaluate for other opportunistic infections including tuberculosis and start ART as soon as possible

ART, antiretroviral treatment; CrAg, cryptococcal antigen; LP, lumbar puncture.

### Detailed recommendations

#### Who to screen?

HIV-seropositive adults and adolescents (≥ 10 years) with a CD4 count < 200 cells/µL are recommended to be screened for cryptococcal antigenaemia. If screening is initiated by a clinician (medical practitioner or a nurse trained to initiate ART) and not performed reflexively in the laboratory, the expert panel recommends that screening must be restricted to adults and adolescents initiating ART for the first time, switching after treatment failure or re-initiating ART (after an episode of interruption that was > 3 months) with a CD4 count < 200 cells/µL and no prior CM. Although adults or adolescents with a CD4 count < 200 cells/µL who are virally suppressed on ART may also be at risk of cryptococcal disease,^14^ there is insufficient current evidence to routinely recommend screening in this group. There are also insufficient data to recommend routine cryptococcal screening of HIV-infected children (< 10 years) in whom the incidence of CM is much lower.^15^ Even though data for adolescents are limited, the WHO currently recommends screening for adolescents. South African data indicate an increasing incidence of CM from the age of 10 years onwards.^15^

#### Screening strategies

With reflexive laboratory CrAg screening, remnant blood samples (submitted to the laboratory for CD4 testing) are tested automatically for CrAg below a specified CD4 threshold. This approach is superior to clinician-initiated screening (where clinicians specifically request a blood CrAg test) in terms of screening coverage.^16^ A South African modelling study comparing the two strategies and using a CD4 threshold of < 100 cells/µL demonstrated that reflex screening was more cost-effective and saved more lives.^7^ Although the CrAg latex agglutination (LA) test format has been more extensively evaluated for the diagnosis of cryptococcal disease, a rapid CrAg lateral flow assay is simpler to perform on blood samples and more accurate.^17^ The National Health Laboratory Service (NHLS) expanded its reflex laboratory CrAg screening service across South Africa in October 2016.^6^ Between October 2016 and March 2019, more than 600 000 persons were screened, with a CrAg+ prevalence of 5.7% (N.P. Govender, pers. comm., 02 September 2019, National Institute for Communicable Diseases). However, the real-world effectiveness of this screen-and-treat strategy in terms of impact on mortality has yet to be determined. The laboratory turnaround time is very short for both CD4 count and reflex CrAg screening test results; however, initiation of ART should not be unnecessarily delayed while awaiting CrAg test results in patients with advanced HIV disease who have no clinical features of meningitis.

#### Management of cryptococcal antigen-positive patients without previous cryptococcal meningitis

All patients who screen CrAg-positive for the first time should have an LP performed. The absence of symptoms of meningitis does not exclude CM: approximately one in three patients with asymptomatic cryptococcal antigenaemia has concurrent CM.^18^ CrAg-positive patients who are subsequently identified as having CM should be managed as per Recommendation 3. In the latter group, ART should be commenced 4–6 weeks after the introduction of antifungal therapy. Patients in whom CM is ruled out by LP (a negative CSF CrAg test is the most rapid method to establish this) should be given oral fluconazole alone as induction therapy (adults: 1200 mg for 2 weeks). This is followed by standard consolidation and maintenance treatment regimens as per Recommendation 3. In these blood CrAg-positive/CSF CrAg-negative patients, ART may be commenced immediately, with the caveat that there is no published evidence for this recommendation.^19^ Adolescents and children should receive fluconazole 12 mg/kg/day (to a maximum of 800 mg per day) for 2 weeks followed by standard consolidation and maintenance treatment. The timing of ART initiation should be the same as adults.

**FIGURE 1:**
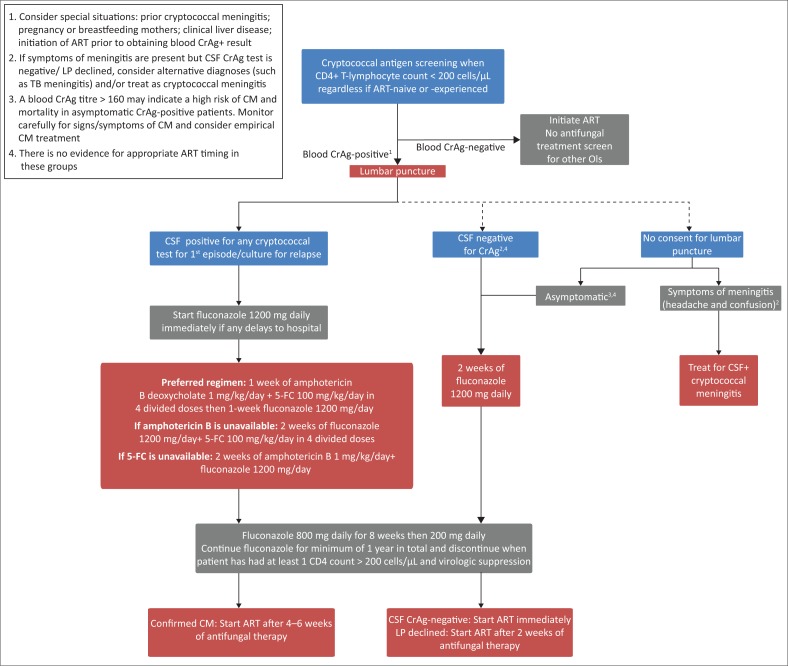
Cryptococcal antigen screening and treatment algorithm.

Patients who decline an LP can be stratified according to the presence or absence of meningitis symptoms (Figure 1), although this approach is now recognised to be suboptimal.^18^ Patients with headache, nausea, vomiting or other signs of CM should be treated as such (as per Recommendation 3), whereas those without symptoms can be treated with fluconazole alone, as for patients in whom CM has been excluded. However, this approach will result in some cases of asymptomatic or subclinical CM being missed. If a screening blood CrAg titre is available (this is not routinely performed in South Africa’s national CrAg screening programme), patients with CrAg titres > 160 may be considered at high risk of CM or cryptococcal disease-related death.^20,21^ Such patients should be carefully monitored for CM signs/symptoms or considered for empirical CM treatment. Community adherence support is recommended for all patients who screen CrAg-positive and are followed up as outpatients, particularly among those who decline an LP.^5^

#### Management of a positive blood cryptococcal antigen result obtained after antiretroviral treatment initiation

Current same-day HIV test-and-treat strategies mean that some patients may start ART before a positive reflex blood CrAg result is received. Given the increased risk of mortality associated with early ART in CM,^22^ the panel recommends immediate referral for LP and CSF analysis in all patients with a positive blood CrAg test who initiated ART in the 4 weeks prior to the CrAg test. Among patients with a negative CSF CrAg test (i.e. in whom CM is excluded), ART can be continued and fluconazole pre-emptive therapy should be initiated. Among patients on a new ART regimen with a positive CSF CrAg test (i.e. a new diagnosis of CM), data suggest that mortality may be increased if CM is diagnosed in the first 4 weeks of ART, possibly mediated through a mechanism of unmasking immune reconstitution inflammatory syndrome (IRIS).^23^ Not all studies have demonstrated this and we do not know if ART interruption would reduce this mortality risk. Potential harms of ART interruption include HIV resistance (more likely with NNRTI-based regimens but less likely with dolutegravir-based regimens) and the risk of HIV disease progression and other opportunistic infections. The panel thought that there was clinical equipoise for the decision whether to continue or stop ART in this group, and we therefore do not, at this stage, recommend ART interruption. This remains a research question.

#### Prior cryptococcal meningitis

Patients with a history of CM do not need to be routinely screened. Cerebrospinal fluid and blood specimens may remain CrAg-positive for months to years after a CM diagnosis and successful treatment of cryptococcal disease and therefore if these tests are positive in the absence of symptoms and signs, this is not an indication of relapse. However, if a patient with prior CM is screened, found to be CrAg-positive and has new symptoms or signs of meningitis, a full evaluation should be undertaken for relapse disease (refer to Recommendations 2 and 7). If the patient does not have new symptoms or signs of meningitis, the clinician should ensure that the patient receives adequate fluconazole maintenance therapy (refer to Recommendation 3).

#### Pregnancy or breastfeeding mothers

Cryptococcal antigen-positive patients who are pregnant should be offered an LP before a decision is made regarding management. If the patient has laboratory evidence of CM, she should be treated for CM with standard treatment.^24,25^ In a pregnant CrAg-positive patient without laboratory-confirmed CM or an asymptomatic woman who declines an LP, the patient should be counselled regarding the risks and benefits of fluconazole treatment. Fluconazole has rarely been associated with human teratogenicity when administered in the first trimester, particularly at higher doses (≥ 400 mg/day). However, patients with a positive blood CrAg test treated with ART and no fluconazole have a substantial risk of progression to meningitis, which has a high mortality. The panel therefore recommends that pregnant women should be counselled that the benefits of fluconazole outweigh the risks in this situation. An option that could be considered in women who are in the first trimester of pregnancy is to treat with fluconazole 200 mg daily until the second trimester. All women exposed to fluconazole in the first trimester should be referred for a high-resolution ultrasound scan before 20 weeks of gestation to detect congenital abnormalities. For mothers who are breastfeeding, consultation with an experienced medical practitioner is also recommended as fluconazole is present at concentrations similar to maternal plasma concentrations in breast milk and can be transmitted in large amounts through breast milk to the infant.^26^

#### Clinical liver disease

Patients with a history of liver disease or with evidence of clinical liver disease deserve careful monitoring of serum liver enzyme and bilirubin levels (with specialist referral if there is a rising trend, or if jaundice develops) because fluconazole may cause liver injury.^27^ Consultation with a medical practitioner experienced in the care of HIV-seropositive patients is recommended.

## Recommendation 2: Laboratory diagnosis and monitoring

### Background

*Cryptococcus neoformans* is the most commonly detected pathogen causing meningitis in South Africa.^28^ All HIV-seropositive adults and adolescents with clinically suspected meningitis or a positive blood CrAg test should be investigated for CM. HIV-seropositive children aged < 5 years are considered to have advanced HIV and, if symptomatic, should also be investigated for CM.^3^ Patients with CM usually seek care with symptoms and signs related to inflamed meninges (including neck stiffness), raised intracranial pressure (including headache, confusion, altered level of consciousness, sixth cranial nerve palsies with diplopia and visual impairment and papilloedema) and encephalitis (including memory loss and new-onset psychiatric symptoms).^29^ Cutaneous lesions and pulmonary involvement (including cavitation, nodular infiltrates and consolidation) may also occur. Symptomatic relapses are common and are most often because of inadequate or premature cessation of maintenance fluconazole treatment.^30^ The incidence of CM is much lower among children^15^; children with CM may present with an acute onset of illness and focal neurological signs may be less common (Table 2).

**TABLE 2:** Summary of recommendation 2.

Scenario	Sub-recommendations
Diagnosis of first episode of suspected CM	All HIV-seropositive adults and adolescents with clinically suspected meningitis or a positive blood CrAg test should be investigated for CM. HIV-seropositive children aged < 5 years are considered to have advanced HIV and, if symptomatic, should also be investigated for CM. An LP should be performed to obtain CSF which should be submitted to a laboratory for a CrAg test and fungal culture. If laboratory facilities are unavailable, a CrAg lateral flow assay may be performed at the bedside on CSF. If opening pressure was not measured at the time of diagnostic LP, an LP should be repeated to measure the pressure once a diagnosis of CM is confirmed – refer to Recommendation 6. We do not recommend baseline CSF CrAg titre or antifungal susceptibility testing.
Diagnosis of CM if LP is not immediately available or if focal neurological signs are present	Serum/plasma/finger prick whole blood may be tested for CrAg to determine if the patient has disseminated cryptococcal disease. Patients with a positive blood CrAg test and symptoms/signs of meningitis should be empirically started on antifungal treatment (Figure 1, Recommendation 3) and referred to a centre where LP can be performed. A CT brain scan should be obtained if there are neurological contraindications to immediate LP.
Diagnosis of subsequent episode of suspected CM	The patient should be assessed clinically for signs and symptoms of meningitis. An LP should be performed to obtain CSF which should be submitted to a laboratory for prolonged fungal culture (minimum 14 days) (Note – India ink and CrAg tests are not useful for the diagnosis of subsequent episodes of cryptococcal meningitis as they can stay positive for a prolonged period despite successful treatment). Opening pressure should be measured. Antifungal susceptibility testing should be considered if the CSF fungal culture is positive and other causes for symptomatic relapse are excluded.
Monitoring response to antifungal treatment	Resolution of symptoms and signs can be used to monitor response to treatment. Unless there is a specific indication (e.g. persistent symptoms or signs suggesting ongoing or late-onset raised intracranial pressure), LP should not be routinely performed after 7–14 days of antifungal treatment to document conversion of CSF from culture-positive to culture-negative.† CSF and serum/plasma CrAg titres should not be monitored.
Suspected antifungal-resistant isolate	Consider antifungal susceptibility testing if a patient has a relapse episode and the causes listed in Table 10 have been excluded (Recommendation 7). Fluconazole MICs should be determined at an academic/reference laboratory and interpreted by an experienced clinical microbiologist in conjunction with clinical findings.
Screening for cryptococcal antigenaemia	Refer to Recommendation 1.

CM, cryptococcal meningitis; LP, lumbar puncture; CSF, cerebrospinal fluid; CrAg, cryptococcal antigen; LFA, lateral flow assay; MIC, minimum inhibitory concentration; CT, computed tomography.

† , If symptoms re-appear or persist during induction treatment, LP should be repeated to re-measure the opening pressure, which may increase despite successful CSF sterilisation – refer to Recommendation 6.

### Detailed recommendations

#### Diagnosis of a first episode of cryptococcal meningitis

An LP is required to confirm the diagnosis and establish the aetiology of suspected meningitis. Lumbar puncture may also alleviate symptoms that are a direct result of raised intracranial pressure. For a suspected first episode of CM, CSF should be submitted to the laboratory for a CrAg test and fungal culture. India ink test is not recommended as the only rapid test because of its lower sensitivity (78% compared to CSF CrAg test).^31^ If a CSF India ink test is performed and found negative, the laboratory should either perform a CSF CrAg test or refer the specimen for this test. A CrAg LFA is the preferred format of testing; there are now several kits on the market although the innovator product (IMMY, Norman, OK) has been most widely studied in pre-clinical and clinical evaluations,^5,17,32^ and the accuracy of other assays is still unclear. *Cryptococcus neoformans* can be cultured from CSF within 72 h among patients with a first episode of CM. *Cryptococcus gattii* is occasionally confirmed on culture (2% of all cases in South Africa) and these infections should be managed as for *C. neoformans* among HIV-seropositive patients.^33^ There is no need to routinely order a baseline CSF CrAg titre because most patients are diagnosed when the CSF fungal burden is already high and the antifungal regimens for a first episode are standardised and not influenced by the CrAg titre (refer to Recommendation 3). A point-of-care CrAg LFA may be performed on CSF at the bedside if laboratory facilities are not immediately available.^34^ Antifungal susceptibility testing should not be routinely requested for a first episode because the vast majority of antifungal minimum inhibitory concentrations (MICs) are low at first diagnosis and, even if elevated, the relevance is difficult to interpret in this setting.^35^ If opening pressure was not measured at the time of diagnostic LP, an LP should be repeated to measure the pressure once a diagnosis of CM is confirmed (refer to Recommendation 6). Cerebrospinal fluid obtained from therapeutic LPs during the course of a hospital admission should not be routinely submitted for laboratory analysis because this is a waste of resources and does not impact on treatment management.

#### Diagnosis of cryptococcal meningitis if focal neurological signs are present or if lumbar puncture is not immediately available

Focal neurological signs are relatively uncommon in CM, except for a sixth cranial nerve palsy. Where focal neurological signs are present, a computed tomography (CT) brain scan should be performed before LP to exclude the presence of space-occupying lesions. If a CT brain scan cannot be performed immediately in the case of focal neurological signs or if an LP is not immediately available to make a diagnosis of meningitis, serum/plasma/whole blood may be tested for CrAg to determine if the patient has disseminated cryptococcal disease. Patients with a positive blood CrAg test *and* symptoms and signs of meningitis are very likely to have CM and should be started empirically on antifungal treatment (refer to Recommendation 3). Patients without focal neurological signs should then be referred to a centre where LP can be performed, while patients with focal neurological signs need to have a CT brain scan performed first followed by an LP (if this is not contraindicated by CT brain findings). Although the expert panel is aware that it may be difficult to access a CT brain scan in rural settings, the panel recommends that there should be urgent referral for a CT scan before LP in blood CrAg-positive patients with focal neurologic signs, whenever possible.

#### Diagnosis of a subsequent episode of cryptococcal meningitis

A careful history should be taken including dates of previous episodes of CM and the patient should be assessed clinically for signs and symptoms of meningitis. An LP is indicated if the patient has signs and symptoms of meningitis. Cerebrospinal fluid should be submitted for fungal culture, with plates incubated for at least 14 days to detect slow fungal growth. Rapid tests are not useful for the diagnosis of subsequent episodes because both CSF India ink and CSF/blood CrAg tests may remain positive for months to years even if treatment has been successful. Antifungal susceptibility testing may be considered for a first relapse episode (see below and refer to Recommendation 7).

#### Monitoring response to treatment

Resolution of symptoms and signs should be used to monitor the response to treatment. An LP should not be routinely performed after 7 (or 14) days of induction antifungal treatment to document conversion of CSF from culture-positive to culture-negative because the expert panel advises routinely changing from induction to consolidation phase treatment at 14 days. Given that a fungal culture result may take up to 14 days to become available, the culture result will not affect the timing of this change. If symptoms persist or recur during induction or at day 7 or 14, an LP should be repeated to re-measure the opening pressure, which may increase despite successful CSF sterilisation. There is no evidence to support extending the duration of induction treatment. The patient should be investigated for other causes of a poor clinical response, the most common being raised intracranial pressure. Patients with raised intracranial pressure should be managed according to Recommendation 6. Daily therapeutic lumbar puncture, and a CT brain scan if possible, is advised. If the cause of a poor clinical response cannot be found, consider referral to a secondary or tertiary centre for review unless the patient’s prognosis is already deemed to be very poor. Cerebrospinal fluid CrAg tests may remain positive for months to years and CrAg titres are not recommended to be routinely measured to monitor the response to treatment. Blood CrAg titres are also not useful to monitor the response to treatment for both CM and asymptomatic antigenaemia.^36,37^

#### Suspected antifungal-resistant isolates

Antifungal susceptibility testing should be considered if the patient has a relapse episode and the causes listed in Table 10 have been excluded. Isolates with elevated fluconazole MICs have been described from relapse episodes – especially where fluconazole monotherapy is initially given – and are unusual if amphotericin B-based induction treatment was administered during the first episode.^35,38^ Because there are no established clinical breakpoints for *C. neoformans* and fluconazole, it is useful to test isolates from the initial and subsequent episodes in parallel in an academic or reference laboratory and document a fourfold (double dilution) change in MIC that may suggest resistance.^25^ This requires that the initial isolate is stored, which may not always be possible at a diagnostic laboratory. Minimum inhibitory concentrations should be interpreted by an experienced clinical microbiologist in conjunction with the clinical history. In the absence of paired isolates, epidemiologic cut-off values can be applied to distinguish wild-type and mutant strains.^39^ Refer to Recommendation 7 for the management of patients with fluconazole-resistant isolates. Non-susceptibility to amphotericin B is very unusual and susceptibility testing for this antifungal agent should not be performed. Flucytosine resistance develops with monotherapy; hence, combination treatment is always recommended.^40^ Baseline flucytosine MIC testing is not routinely recommended at present.

## Recommendation 3: Management of a first episode of cryptococcal meningitis

### Detailed recommendations

The antifungal treatment of HIV-associated CM is divided into three phases: induction, consolidation and maintenance (Table 3).

**TABLE 3:** Summary of recommendation 3.

Phase	Duration	Treatment
Induction	2 weeks	*Preferred regimen*: 1 week of amphotericin B deoxycholate (1 mg/kg/day) and flucytosine (100 mg/kg/day divided into four doses per day), followed by 1 week of fluconazole (1200 mg daily for adults; 12 mg/kg/day for children and adolescents up to a maximum of 800 mg daily) Alternative options 2 weeks of fluconazole (1200 mg daily for adults; 12 mg/kg/day for children and adolescents) and flucytosine (100 mg/kg/day divided into four doses per day) Preferred option if flucytosine is unavailable: 2 weeks of amphotericin B deoxycholate (1 mg/kg/day) and fluconazole (1200 mg daily for adults, 12 mg/kg/day for children and adolescents) If liposomal amphotericin B is available for patients with renal dysfunction: 1 week of liposomal amphotericin B (3 mg/kg/day – 4 mg/kg/day) and flucytosine (100 mg/kg/day divided into four doses per day), followed by 1 week of fluconazole (both fluconazole and flucytosine doses adjusted for renal impairment)
Consolidation	8 weeks	Fluconazole (800 mg daily for adults; 12 mg/kg/day for children and adolescents up to a maximum of 800 mg daily)
Maintenance	Continue for at least 12 months until a single CD4 count > 200 cells/µL and a suppressed HIV viral load	Fluconazole (200 mg daily for adults; 6 mg/kg/day for children and adolescents up to a maximum of 200 mg daily)

#### Induction phase (2 weeks)

The WHO recommends that the first choice for induction phase treatment is amphotericin B 1 mg/kg/day and flucytosine 100 mg/kg/day divided into four doses per day.^4^ Flucytosine is not currently registered in South Africa or any other African country.^10,41^ A flucytosine product manufactured in Poland was acquired by Mylan in 2016. Despite previously lapsed registration in South Africa and approval by the WHO Pre-Qualification Program, the South African Health Products Regulatory Authority (SAHPRA) requires a full dossier to be submitted. The panel and the Southern African HIV Clinicians Society support advocacy efforts to provide greater access to flucytosine through fast-track SAHPRA registration and development of additional generic products, particularly in light of the findings of the recently published Advancing Cryptococcal Treatment in Africa (ACTA) trial.^8,10,41,42^ This trial demonstrated a substantial mortality reduction among participants randomised to flucytosine-based combination therapies (Table 4). Overall, the best-performing treatment arm in the ACTA trial was the 1-week combination of amphotericin B deoxycholate and flucytosine, followed by high-dose oral fluconazole in the second week. This regimen was also associated with fewer adverse effects, specifically a reduction in amphotericin B deoxycholate-related nephrotoxicity and anaemia, and lower costs. This is the preferred regimen recommended by the panel. Both the all-oral fluconazole/flucytosine regimen and 1-week amphotericin B deoxycholate/flucytosine regimen were non-inferior to the previous gold standard (i.e. 2 weeks of amphotericin B and flucytosine) in terms of 2-week mortality. The all-oral regimen is useful in cases where a delay in administration of amphotericin B deoxycholate is expected or its use is contraindicated. The panel now also recommends a higher induction dose of fluconazole (1200 mg daily) based on doses used in the ACTA trial. There is no evidence from fluconazole monotherapy trials to suggest an increased toxicity at this higher dose.^43,44^ If flucytosine is unavailable, the preferred treatment option is 2 weeks of amphotericin B and fluconazole (not 1 week of amphotericin B and fluconazole) because the 2-week regimen was associated with lower 2- and 10-week mortality rates than 1 week of this drug combination in the ACTA trial (Table 4). In settings with access to liposomal amphotericin B, this antifungal may be used instead of the deoxycholate formulation, especially among patients with renal impairment. Although there is no evidence of improved efficacy compared to amphotericin B deoxycholate, liposomal amphotericin B is associated with fewer adverse effects and may be available at a reduced cost for CM in some countries.^45,46^ In countries where amphotericin B is unavailable, the panel advises clinicians to follow the WHO guideline with respect to high-dose fluconazole options.^4^ In South Africa, all patients with CM should be treated with an amphotericin B-based induction regimen unless there is a specific contraindication to this.

**TABLE 4:** Unadjusted 2- and 10-week mortality outcomes for the five combination treatment regimens in the Advancing Cryptococcal Treatment in Africa trial.

Regimen	2-week mortality			10-week mortality		
	*n*/*N*	%	95% CI	*n/N*	%	95% CI
One-week amphotericin B and flucytosine (short course)	13/113	11.6	5.7–17.5	27/113	24.2	16.2–32.1
Two-week fluconazole and flucytosine (all-oral regimen)	41/225	18.2	13.2–23.3	79/225	35.1	28.9–41.3
Two-week amphotericin B and flucytosine	24/115	20.9	13.4–28.3	44/115	38.3	29.4–47.2
Two-week amphotericin B and fluconazole	25/114	21.9	14.3–29.5	47/114	41.3	32.3–50.4
One-week amphotericin B and fluconazole	36/111	32.4	23.7–41.1	54/111	48.6	39.4–57.9

CI, confidence interval; *n*/*N*, number of deaths divided by number of participants in that trial arm.

#### Consolidation phase (further 8 weeks)

The panel recommends a fluconazole dose of 800 mg daily for 8 weeks. This recommendation is based on expert opinion.

#### Maintenance phase (secondary prophylaxis)

The panel recommends fluconazole 200 mg daily for at least 10 more months (i.e. to complete a total of 12 months of antifungal treatment). Maintenance fluconazole should only be stopped when the CD4 count is documented to be > 200 cells/µL (on one occasion is sufficient) and the most recent HIV viral load is suppressed.

#### Adolescents and children

During the induction phase, amphotericin B should be prescribed at a daily dose of 1 mg/kg/day and flucytosine as a (divided) daily dose of 100 mg/kg/day (Table 5). Fluconazole doses should also be calculated by body weight: induction phase: 12 mg/kg/day (up to 800 mg daily); consolidation phase: 6 mg/kg/day – 12 mg/kg/day (up to 800 mg daily); and maintenance phase: 6 mg/kg/day (up to 200 mg daily).

**TABLE 5:** Flucytosine dosing in children and adults with normal renal function.

Lower weight limit (kg)	Upper weight limit (kg)	Number of pills	Total dose (mg)	Daily dose for lower weight limit (mg/kg)	Daily dose for upper weight limit (mg/kg)	Dose 1†	Dose 2†	Dose 3†	Dose 4†
20	24	4	2000	100.00	83.33	1	1	1	1
25	29	5	2500	100.00	86.21	2	1	1	1
30	34	6	3000	100.00	88.24	2	1	2	1
35	39	7	3500	100.00	89.74	2	2	2	1
40	44	8	4000	100.00	90.91	2	2	2	2
45	49	9	4500	100.00	91.84	3	2	2	2
50	54	10	5000	100.00	92.59	3	2	3	2
55	59	11	5500	100.00	93.22	3	3	3	2
60	64	12	6000	100.00	93.75	3	3	3	3
65	69	13	6500	100.00	94.20	4	3	3	3
70	74	14	7000	100.00	94.59	4	3	4	3
75	79	15	7500	100.00	94.94	4	4	4	3
80	84	16	8000	100.00	95.24	4	4	4	4

† , Number of 500 mg pills per dose.

#### Renal impairment (creatinine clearance < 50 mL/min)

If there is renal impairment at the time of diagnosis of CM with a calculated creatinine clearance of 30 mL/min – 50 mL/min, the recommended induction regimens are the following:

- If liposomal amphotericin B is available, switch to 1 week of liposomal amphotericin B (3 mg/kg/day – 4 mg/kg/day) and flucytosine, followed by 1 week of fluconazole (both fluconazole and flucytosine dose adjusted according to creatinine clearance) (Table 6).
- One week of amphotericin B deoxycholate (1 mg/kg/day) and flucytosine (dose adjusted according to creatinine clearance), followed by 1 week of fluconazole (dose adjusted according to creatinine clearance) (Table 6).

**TABLE 6:** Induction therapy doses of flucytosine, fluconazole and amphotericin B adjusted according to estimated glomerular filtration rates for adults.

Antifungal agent	eGFR > 50	eGFR 10–50	eGFR < 10	Haemodialysis
Amphotericin B deoxycholate	1 mg/kg	1 mg/kg	1 mg/kg	1 mg/kg (can administer during dialysis)
Fluconazole	1200 mg daily	600 mg daily	600 mg daily	600 mg daily; dose after dialysis
Flucytosine	25 mg/kg 6 hourly	25 mg/kg 12 hourly	25 mg/kg daily	25 mg/kg daily; dose after dialysis

*Source:* The Sanford guide to antimicrobial therapy 2019 / editors, David N. Gilbert, M.D., George M. Eliopoulos, M.D., Henry F. Chambers, M.D., Michael S. Saag, M.D., Andrew T. Pavia, M.D. Sperryville, VA, USA: Antimicrobial Therapy, Inc., [2019]

Note: Multiply by 0.85 for women. Children: CrCL 20 mL/min – 40 mL/min: flucytosine 25 mg/kg q12h; 10 mL/min – 20 mL/min: flucytosine 25 mg/kg q24 h; < 10 mL/min: flucytosine 25 mg/kg q24–48h. Glomerular filtration rate (GFR) is a key indicator of renal function. Estimated GFR (eGFR) is a mathematically derived entity based on a patient’s serum creatinine level, age, sex and race. Creatinine clearance (CrCL), an estimate of GFR, can be estimated in adults using the Cockcroft–Gault equation: [[140-age (years)]*weight (kg)] / [0.8136*serum creatinine (µmol/L)].

If there is renal impairment at baseline with a creatinine clearance of < 30 mL/min or a deterioration in renal function, the recommended induction regimens are the following:

- If liposomal amphotericin B is available, 1 week of liposomal amphotericin B (3 mg/kg/day – 4 mg/kg/day) and flucytosine, followed by 1 week of fluconazole (both fluconazole and flucytosine dose adjusted according to creatinine clearance).
- Two weeks of fluconazole (dose adjusted according to creatinine clearance) and flucytosine (dose adjusted according to creatinine clearance).
- If neither flucytosine nor liposomal amphotericin B is available, administer a single dose of amphotericin B deoxycholate (0.7 mg/kg) and daily fluconazole (dose adjusted according to creatinine clearance) and monitor creatinine clearance daily. Administer amphotericin B deoxycholate (0.7 mg/kg) on alternate days if the creatinine clearance remains static and give as many doses as possible over the 2-week induction period.

#### Important practice point

Clinicians should be very clear when prescribing liposomal amphotericin B versus amphotericin B deoxycholate. The daily doses are different.

Intravenous fluids should be administered to all patients. Tenofovir and amphotericin B deoxycholate can be used together provided the patient’s renal function is normal and serum creatinine does not double on treatment. Among patients with renal impairment, ART regimens containing tenofovir should be adjusted, switching to an alternative antiretroviral agent such as abacavir. If the patient’s initial reason for a reduced creatinine clearance was dehydration and this has been corrected with fluid administration, the patient can be switched back to amphotericin B deoxycholate at normal doses. Flucytosine has a short plasma half-life and is cleared unchanged via the kidneys. Flucytosine requires dose adjustment if there is renal impairment; where patients are dialysed, it should be given after dialysis (Table 6). Fluconazole monotherapy is not recommended except as a last resort. When used as monotherapy during induction, the fluconazole dose is 1200 mg daily with normal renal function. With a creatinine clearance of < 50 mL/min, the induction dose of fluconazole should be reduced by 50% to 600 mg daily.

#### Management of other forms of disseminated cryptococcosis

Cryptococcal fungaemia (i.e. a positive blood culture) should be managed as per CM. The treatment of cryptococcomas, pulmonary cryptococcosis and other forms of culture-confirmed disseminated cryptococcal disease is beyond the scope of this guideline. Readers are advised to consult the 2010 Infectious Diseases Society of America guideline.^25^

#### Patients on tuberculosis treatment

The panel does not recommend a fluconazole dose increase among patients receiving rifampicin because the induction of fluconazole metabolism by rifampicin causes only moderate reductions in fluconazole exposure and because of the high doses of fluconazole that are now being recommended for induction and consolidation treatment.^8,47^

#### Adjunctive corticosteroid therapy

The expert panel advises against adjunctive corticosteroid therapy in the initial management of CM.^9^ Refer to Recommendation 7 for the use of corticosteroids among patients with IRIS.

#### Immunological failure on antiretroviral treatment

If patients with priorly treated CM develop immunological failure on ART and their CD4 count drops below 200 cells/µL after secondary prophylaxis has been stopped, the panel advises restarting fluconazole at 200 mg daily. This may be considered for patients with priorly treated cryptococcal antigenaemia too. Refer to the Maintenance phase (secondary prophylaxis) for duration of treatment.

#### Non-adherence to maintenance treatment

Among patients who stop taking fluconazole maintenance prematurely and then return for care but are asymptomatic, the panel advises simply restarting fluconazole 200 mg daily and monitoring closely for recurrence of meningitis. Symptomatic patients should be fully investigated for CM. Community adherence support for ART and fluconazole should be arranged. Refer to the Maintenance phase (secondary prophylaxis) for duration of treatment.

#### Analgesia

Therapeutic LPs are the best form of ‘analgesia’ for headaches associated with raised intracranial pressure. Paracetamol can be used but not non-steroidal anti-inflammatory drugs (NSAIDs) because of the nephrotoxicity concerns with amphotericin B deoxycholate. Morphine may also be appropriate and is not contraindicated in the presence of raised intracranial pressure.

## Recommendation 4: Amphotericin B and flucytosine toxicity prevention, monitoring and management

### Background

#### Amphotericin B deoxycholate-related toxicities

Major adverse effects of amphotericin B deoxycholate include acute kidney injury (AKI) (caused by renal vasoconstriction and acute tubular necrosis) usually in the second week of therapy, hypokalaemia, hypomagnesaemia, anaemia, febrile reactions and chemical phlebitis.^48^ Acute kidney injury and electrolyte abnormalities may be prevented by pre-hydration, by avoiding concurrent use of other nephrotoxins (e.g. NSAIDs and aminoglycosides) and by routine administration of potassium and magnesium supplements. Phlebitis is very common among patients receiving amphotericin B and increases the risk of localised cellulitis as well as sepsis. Anaemia commonly occurs among patients receiving amphotericin B and can be clinically significant particularly among those with a low baseline haemoglobin level. Decreases in haemoglobin of greater than 2 g/dL occurred in 50% – 71% of patients over 2 weeks’ treatment in an individual-level analysis of data from several trials.^49^ It is important to also exclude other treatable causes of anaemia and consider transfusion among symptomatic patients. It should be noted that both the nephrotoxic effect of amphotericin B deoxycholate and the decrease in haemoglobin are less commonly observed when using the preferred regimen of 1-week amphotericin B combined with flucytosine compared to regimens using 2 weeks of amphotericin B (Table 7).^8^

**TABLE 7:** Summary of recommendation 4.

Scenario	Sub-recommendations
Administration of amphotericin B deoxycholate†	Amphotericin B powder should be reconstituted in sterile water; inject the calculated volume of reconstituted antifungal in water into 1 L of 5% dextrose water and administer within 24 h Amphotericin B can be administered via a peripheral intravenous (IV) line if the solution contains ≤ 0.1 mg of amphotericin B per 1 mL of 5% dextrose water A test dose is unnecessary The solution should be infused over at least 4 h
Administration of flucytosine	Flucytosine is available as 500 mg tablets With normal renal function, the dose is 100 mg/kg/day per os in four divided doses Therapeutic monitoring of serum levels is not recommended at this dose
Prevention of amphotericin B deoxycholate-related toxicities	Adults should be pre-hydrated with 1 L of normal saline containing 1 ampoule of potassium chloride (20 mmol) infused over 2 h before the amphotericin B infusion‡ Twice daily oral potassium and daily oral magnesium supplementation should be administered to adults To minimise the risk of phlebitis, lines should be flushed with normal saline immediately after the amphotericin B infusion is complete and the infusion bag should not be left attached to the intravenous administration set after the infusion is complete
Prevention of flucytosine-related toxicity	Drug accumulation and increased risk for toxicity occurs with renal dysfunction. The dose therefore needs to be carefully adjusted according to the estimated glomerular filtration rate
Monitoring of patients receiving amphotericin B and flucytosine	Days 0, 3 and 7: creatinine and potassium (and magnesium, if available) Days 0 and 7: full blood count (with a differential count if available). Day 3: full blood count and differential can be considered when flucytosine is used, especially if baseline abnormalities exist. Flucytosine may cause bone marrow suppression but this is uncommonly observed with short duration of use and the current suggested dosing schedule Fluid input and output monitoring
Management of amphotericin B-related toxicities	Refer to Recommendation 3 (baseline renal impairment section) Febrile reactions can be treated with paracetamol 1 g 30 min before infusion (if severe, hydrocortisone 25 mg IV can be given before subsequent infusions)
Management of flucytosine-related toxicities	If grade 4 neutropenia or if any neutropenia-related complications develop, reduce the flucytosine dose and repeat a neutrophil count immediately. If neutropenia is confirmed, stop the flucytosine and switch to fluconazole. If the patient was being treated with amphotericin B and flucytosine, consider a second week of amphotericin B deoxycholate treatment.

† , For adolescents and children, doses should be calculated by body weight;

‡ , For children and adolescents, normal saline, with 1 ampoule of potassium chloride (20 mmol) added per litre of fluid, should be infused at 10 mL/kg – 15 mL/kg over 2–4 h (not more than 1 L) prior to amphotericin B administration. If saline is unavailable, then other parenteral rehydration solutions, for example, Darrow’s solution or Ringer’s lactate, that already contain potassium can be used.

#### Flucytosine-related toxicities

Flucytosine is associated with bone marrow toxicity and resultant anaemia, neutropenia and thrombocytopenia. This was observed particularly in early studies when flucytosine was used at high doses for prolonged periods of time.^50,51,52^ The reported prevalence of toxicity from recent trials, using lower doses for shorter periods, has been much lower. In the ACTA trial, the preferred regimen of 1-week amphotericin B combined with flucytosine 100 mg/kg/day was not associated with an increase in severe neutropenia compared to flucytosine-free regimens.^8^ The bioavailability of oral flucytosine was reduced among Thai patients with advanced HIV disease compared to the intravenous formulation, minimising toxicity but with no demonstrated difference in early fungicidal activity.^53^ For these reasons, therapeutic monitoring of serum levels is not recommended in the setting of HIV-related CM. Flucytosine plasma clearance is closely related to creatinine clearance and flucytosine thus accumulates with impaired renal function; this may lead to increased risk of toxicity. Close monitoring of renal function and appropriate dose adjustment according to estimated creatinine clearance are therefore essential.^54^

### Detailed recommendations

#### Administration

**Amphotericin B deoxycholate:** Amphotericin B deoxycholate powder (50 mg vials) should be refrigerated between 2 °C and 8 °C and protected from light.^55^ The total daily dose of amphotericin B is calculated based on a dose of 1 mg/kg/day; amphotericin B deoxycholate powder from each 50 mg vial should be aseptically reconstituted in 10 mL of sterile water. The calculated volume of the concentrate (i.e. reconstituted drug in sterile water) should be injected into a 1 L bag of 5% dextrose water and shaken to mix well. Amphotericin B deoxycholate should never be mixed with normal saline or half-normal saline as it will precipitate. Once mixed, the solution, containing ≤ 0.1 mg of amphotericin B per 1 mL of 5% dextrose water for infusion through a peripheral intravenous line, must be infused within 24 h of preparation, or else it should be discarded.^55^ A test dose is not recommended and protection of the solution from light with a brown bag is unnecessary.^55^ The line that is used for amphotericin B infusion should not be used to simultaneously administer other medications. The solution should be infused over 4 h or more (infusion < 4 h can result in cardiac complications). Once the infusion is complete, the line should be flushed immediately with normal saline.

**Flucytosine:** Flucytosine (500 mg tablets) should be stored below 25 °C in a cool, dry area. With normal renal function, the total daily dose is 100 mg/kg/day given in four divided doses (i.e. every 6 h) per os (Table 5). Nausea and vomiting may occur and can be attenuated by administering flucytosine tablets individually during a 15-min window or by pre-medication with anti-emetics.

### Prevention of toxicities

#### Amphotericin B deoxycholate

Patients should be pre-hydrated with 1 L of normal saline containing 1 ampoule of potassium chloride (20 mmol K^+^ per 10 mL ampoule) infused over 2 h before administration of amphotericin B deoxycholate. This reduces renal toxicity and hypokalaemia. Patients should be given 1200 mg potassium chloride orally twice daily (equivalent to 16 mmol of oral potassium, e.g. two Slow-K 600 mg tablets twice daily, 8 mmol K^+^ per tablet) and up to 1500 mg magnesium chloride orally daily (e.g. two Slow-Mag 535 mg tablets daily, 5.33 mmol Mg2+ per tablet, or two Ultimag tablets daily, 660 mg Mg2+ with zinc oxide 6 mg) for the duration of treatment with amphotericin B deoxycholate.^26^ Routine pre-emptive potassium supplementation should not be given to patients with pre-existing renal impairment or hyperkalaemia. To minimise the risk of phlebitis, lines should be flushed with normal saline immediately after amphotericin B infusion is complete. The empty bag should not be left attached to the intravenous line. The intravenous line should be removed if the patient develops a fever after the infusion or at the first sign of redness or discomfort at the insertion site. Febrile reactions may occur; in order to prevent recurrence, the infusion should be administered at a slow rate over the first half hour while observing the patient closely and treatment such as paracetamol may be required.

#### Flucytosine

Renal function should be closely monitored especially when flucytosine is combined with amphotericin B deoxycholate. The dose of flucytosine should be adjusted according to the estimated creatinine clearance in order to prevent accumulation and increased toxicity (Table 6).

### Laboratory or clinical monitoring

Recommendations depend on the induction regimen (Table 8).

**TABLE 8:** Laboratory monitoring according to induction regimen used.

Induction regimen	Week 1	Week 2	Laboratory monitoring
Preferred	Amphotericin B deoxycholate + 5-FC	Fluconazole	Day 0: Full blood count and differential, creatinine clearance, potassium, magnesium
			Day 3: Full blood count (only if low baseline haemoglobin), creatinine clearance, potassium, magnesium
			Day 7: Full blood count and differential, creatinine clearance, potassium, magnesium
Amphotericin B unavailable	Fluconazole + 5-FC	Fluconazole + 5-FC	Day 0: Full blood count and differential, creatinine clearance
			Day 3: Full blood count (if low baseline haemoglobin)
			Day 7: Full blood count and differential
			Day 10: Full blood count and differential (if any abnormalities previously)
			Day 14: Full blood count and differential, creatinine clearance can be done more frequently if baseline is abnormal
5-FC is unavailable	Amphotericin B deoxycholate + fluconazole	Amphotericin B deoxycholate + fluconazole	Day 0: Creatinine clearance, potassium, magnesium, full blood count
			Day 3: Creatinine clearance, potassium, magnesium
			Day 7: Creatinine clearance, potassium, magnesium, full blood count
			Day 10: Creatinine clearance, potassium, magnesium
			Day 14: Creatinine clearance, potassium, magnesium, full blood count

#### Amphotericin B deoxycholate

At a minimum, baseline and twice weekly monitoring of serum creatinine/potassium and baseline and weekly monitoring of haemoglobin are recommended for the duration of amphotericin B deoxycholate treatment. Renal toxicity is more likely to develop in the second week of treatment in regimens where amphotericin B is used for 2 weeks. Fluid input and output should be carefully monitored. Chemical phlebitis is often complicated by infection at the intravenous line insertion site, which can result in bacteraemia^56^; the insertion site should be monitored by regular clinical examination and febrile patients with a suspected insertion site infection should be appropriately investigated and managed.

#### Flucytosine

Baseline grade 1 neutropenia (defined as an absolute neutrophil count ≤ 1000 cells/mm^3^) was documented among 12% of participants enrolled into the Cryptococcal Optimal ART Timing and Adjunctive Sertraline for the Treatment of HIV-Associated Cryptococcal Meningitis clinical trials where they received amphotericin B and fluconazole (plus sertraline in some patients).^57^ Patients with baseline grade 1 neutropenia did not have a higher 30-day mortality compared to those without neutropenia. A baseline full blood/differential count should be done where available and the risk–benefit ratio of flucytosine therapy should be considered in the setting of baseline neutropenia. However, we strongly recommend the use of flucytosine even when there is baseline neutropenia because of the substantial mortality benefit over alternative regimens. Both amphotericin B and flucytosine are associated with anaemia and thus serum haemoglobin should be monitored. A full blood count should be done at least weekly. A baseline differential count should be done if available and be repeated with subsequent full blood counts. Creatinine clearance needs to be monitored in order to adjust flucytosine dose. This is particularly important where baseline renal function impairment exists or where flucytosine is administered with amphotericin B deoxycholate (Table 6).

#### Fluconazole

The panel recommends checking alanine transaminase (ALT) levels if symptoms of hepatitis or jaundice develop while patients are on fluconazole, but routine ALT monitoring is not indicated.

### Management of toxicities

#### Amphotericin B deoxycholate

For significant hypokalaemia (serum K^+^ <3.3 mmol/L), additional intravenous replacement is required: up to 2 ampoules of potassium chloride (20 mmol K+ per 10 mL ampoule) in 1 L of normal saline 8 hourly. Among those who develop hypokalaemia, serum potassium should be monitored daily until it is resolved. If hypokalaemia remains uncorrected, serum magnesium should be checked (if this test is available) and/or oral magnesium supplementation should be doubled. Intravenous magnesium sulphate may be considered for persistent hypokalaemia and hypomagnesaemia. If serum creatinine doubles from baseline, one dose of amphotericin B deoxycholate may be omitted and/or pre-hydration may be increased to 1 L of normal saline 8 hourly; serum creatinine should then be monitored daily. If serum creatinine improves, amphotericin B may be restarted at a dose of 0.7 mg/kg/day and/or alternate-day treatment could be considered. If creatinine remains elevated or repeatedly rises, liposomal amphotericin B should be substituted if available or amphotericin B deoxycholate should be stopped and an alternative regimen should be used (refer to Recommendation 3). If febrile reactions occur with amphotericin B, paracetamol 1 g may be given 30 min before infusion or, for severe reactions, hydrocortisone 25 mg IV can be administered before subsequent infusions.^22^

#### Flucytosine

Bone marrow suppression is uncommon at the recommended induction flucytosine dose for 1 week only, especially if renal function is normal (dose adjustment is important in case of significant renal impairment). Other causes of a low neutrophil count should also be considered. However, if grade 4 neutropenia (< 400 cells/mm^3^) develops, then we recommend to reduce the flucytosine dose and repeat a neutrophil count as soon as possible. If the neutropenia is confirmed, then withhold flucytosine until counts recover. If the patient was on the preferred 1-week amphotericin B and flucytosine regimen, fluconazole may be added to amphotericin B, and extension of amphotericin B treatment to a second week should be considered.

## Recommendation 5: Timing of antiretroviral therapy

### Detailed recommendations

#### Cryptococcal meningitis

The expert panel recommends commencing ART 4–6 weeks after the diagnosis of CM. The panel strongly advises that ART must not be delayed beyond 6 weeks after diagnosis, and most members of the panel advise that clinicians should aim to start exactly 4 weeks after diagnosis of CM. Although most patients with CM have advanced immunosuppression with very low CD4 counts, two randomised clinical trials conducted in sub-Saharan Africa have shown excess early mortality when ART was commenced while patients were still receiving induction phase treatment for CM.^22,58^ In the later trial conducted in Uganda and South Africa, patients who started ART 1–2 weeks after diagnosis of CM had a 15% higher mortality than those who deferred ART until 5–6 weeks.^59^ Another small trial showed possible excess IRIS in those patients who started ART early.^60^ The in-hospital stay associated with amphotericin B therapy should be used for pre-ART counselling, identification of a treatment supporter and early referral to an ART clinic. Clinicians should aim to set up an ART clinic appointment within a week of discharge from hospital to prevent delays in ART initiation beyond what is advised in this guideline. Patients initiated on ART should be counselled regarding the risk of development of IRIS. If a patient is referred to another facility for ART, the need for fluconazole consolidation or maintenance therapy should be communicated. The expert panel recommends standard first-line ART regimens among patients with CM.^12^ If nephrotoxicity occurred on amphotericin B, the renal function should be checked before starting ART to ensure that it has improved (to a creatinine clearance of > 60 mL/min) before commencing tenofovir. The panel advises that, among patients who present with relapse of CM or a first CM episode after interrupting ART, ART should also be restarted after 4–6 weeks. One situation where ART may be delayed further is if a patient is still symptomatic with headaches at the visit when ART is about to be started. In such a situation, a LP should be repeated to measure pressure and for fungal culture to exclude persistent culture positivity. Antiretroviral treatment should be deferred and such patients may require further LPs or amphotericin B to ensure control of symptoms before starting ART (Table 9).

**TABLE 9:** Summary of recommendation 5.

Scenario	Sub-recommendations
Following a first or relapse episode of CM	Start ART 4–6 weeks after diagnosis of CM. The panel strongly advises that ART must not be delayed beyond 6 weeks after diagnosis, and most members of the panel advise that clinicians should aim to start exactly 4 weeks after diagnosis of CM No adjustment in first-line ART regimen is required for patients who are ART-naïve (unless renal dysfunction precludes the use of tenofovir)
Following a new diagnosis of cryptococcal antigenaemia	If CM has been excluded, start ART immediately Asymptomatic CrAg-positive patients who decline consent for LP or for whom LP is contraindicated should start on ART after at least 2 weeks of antifungal treatment

CM, cryptococcal meningitis; LP, lumbar puncture; ART, antiretroviral treatment.

#### Cryptococcal antigenaemia

The panel advises starting ART immediately among ART-naïve patients who are blood CrAg-positive on screening and have an LP that excludes CM (with a caveat that there is no evidence for this recommendation). Asymptomatic CrAg-positive patients who decline consent for LP or for whom LP is contraindicated should start ART after at least 2 weeks of antifungal treatment (Figure 1).

## Recommendation 6: Management of raised intracranial pressure

### Background

Raised intracranial pressure occurs in up to 75% of patients with CM and results from physical obstruction of CSF outflow through the arachnoid villi/granulations resulting in build-up of CSF pressure.^61,62^ Raised pressure may be present at the diagnosis of CM or develop while the patient is on treatment and may result in severe headaches, vomiting, confusion or depressed level of consciousness, ophthalmoplegia (particularly sixth cranial nerve palsies) and visual disturbance/loss. Clinicians need to consider raised intracranial pressure and manage appropriately if a patient exhibits these symptoms or signs at any stage of the management of CM. To alleviate raised pressure, therapeutic LPs are indicated. New-onset hypertension may be a sign of increased intracranial pressure (i.e. part of Cushing’s triad) and should prompt an LP to measure opening pressure instead of anti-hypertensive medications (Box 1).

BOX 1 Summary of recommendation 6.

**Sub-recommendations**

Measure baseline opening pressure at the time of or shortly after the diagnosis of CM using a manometer

If opening pressure is > 25 cm H_2_O (manometer reading), remove 10 mL – 30 mL CSF

Repeat LP whenever there are symptoms or signs of raised intracranial pressure (headache, vomiting, drowsiness, confusion, sixth cranial nerve palsy, visual disturbance, etc.)

Daily therapeutic LPs may be required

CM, cryptococcal meningitis; CSF, cerebrospinal fluid; LP, lumbar puncture.

### Detailed recommendations

#### Measurement of opening pressure

The panel agreed that it is a good practice to measure the CSF opening pressure whenever a diagnostic LP is performed. However, in practice, the opening pressure may not have been measured at the initial diagnostic LP. Thus, once the diagnosis of CM is made, an LP should be repeated to measure CSF opening pressure, particularly if the patient still has a headache (which is usually the case). The pressure should be measured using a manometer with the patient lying down and without excessive spinal flexion. Approximately 15% of patients with initially normal intracranial pressure will develop raised intracranial pressure during treatment; thus, all patients should be monitored daily for headache or signs of raised intracranial pressure that should prompt an LP.

#### What to do if a manometer is unavailable?

Manometers are not readily available in all centres or settings. In the absence of a manometer, the CSF pressure can be crudely estimated in various ways:

- Drop counting: obtaining ≥ 40 drops of free-flowing CSF in 60 s using a 22-gauge spinal needle suggests a high CSF pressure.^63^
- An ‘eyeball test’: a powerful squirt of CSF from the LP needle suggests a high CSF pressure.
- Makeshift manometers from intravenous line sets can be used to estimate opening pressure in cm H_2_O although these sets consistently under-estimate the opening pressure.^64^

The panel cautions that the above methods may be prone to under-estimating the actual CSF pressure based on a manometer reading.

#### Management of raised pressure

If the opening pressure is raised (i.e. a manometer reading > 25 cm H_2_O), then 10 mL – 30 mL CSF should be drained (to normalise pressure **to** < 20 cm H_2_O or decrease the pressure by at least 50% – based on repeat measurement of closing pressure). Then the need for pressure relief should be dictated by recurrence of symptoms of raised intracranial pressure. Patients may require daily LPs. Where a manometer is not available and there are clinical symptoms or signs of raised intracranial pressure, we advise performing an LP and removing 20 mL – 30 mL of CSF. A symptomatic improvement after the therapeutic LP would support the symptoms having been due to raised intracranial pressure (patients with raised intracranial pressure typically experience considerable relief of symptoms following a therapeutic LP). The patient should then be reviewed on subsequent days for ongoing symptoms and signs of raised intracranial pressure, which would indicate the need for further therapeutic LPs.

In rare cases, twice daily LPs are required for controlling raised intracranial pressure in patients with extremely high opening pressures. Patients with persistent pressure symptoms and measured high opening pressures who fail to respond to daily LPs for more than 1 week may require lumbar drain insertion or shunting procedures. In such cases, neurosurgical consultation should be sought.

## Recommendation 7: Management of relapse episodes of cryptococcal meningitis

There are several possible reasons for recurrence of symptoms of meningitis among patients treated for CM. In certain cases, recurrence is caused by microbiological relapse (although this is very uncommon with good adherence to maintenance fluconazole). There are situations in which there is a recurrence of symptoms but CSF fungal cultures are negative. The causes are summarised in Table 10.

**TABLE 10:** Possible causes of recurrent symptoms and signs of meningitis in cryptococcal meningitis.

Symptoms	Causes
**Attributable to CM**	
CM relapse†	Possible causes of CM relapse (positive fungal culture) Fungal: Inadequate induction therapy (e.g. suboptimal amphotericin B deoxycholate administration because of toxicity) Non-adherence to fluconazole consolidation or maintenance therapy Fluconazole resistance (uncommon if preferred induction regimens are used) CNS cryptococcomas or gelatinous pseudocysts (requiring prolonged induction therapy) Immunological: ART not initiated 4–6 weeks after CM induction therapy Immunological failure because of virological failure of ART
Paradoxical IRIS	Features of IRIS (most cases have negative CSF fungal culture) Occurs weeks to months after ART initiation Because of an inflammatory response directed at antigens of non-viable fungus Associated with higher CSF white cell counts, compared to the initial (culture positive) episode of CM Frequently accompanied by raised intracranial pressure and can be associated with focal brain inflammation and/or mass lesions
Persistently elevated ICP	Thought to be mediated by occlusion of arachnoid granulations by fungi and fungal capsule; this does not necessarily imply CM treatment failure.
**Unrelated to CM**	
New diagnosis	Possible causes: Tuberculous meningitis Viral or bacterial meningitis Space-occupying lesion with cerebral oedema (e.g. tuberculoma, CNS malignancy) or hydrocephalus Non-infective (e.g. tension headache)

CM, cryptococcal meningitis; ART, antiretroviral treatment; IRIS, immune reconstitution inflammatory syndrome; CNS, central nervous system; ICP, intracranial pressure.

† , Relapse is defined as recurring clinical features of CM because of recurrent or ongoing *C. neoformans* growth in the CNS, diagnosed on positive CSF fungal culture.

### Initial assessment

When a patient seeks care for a recurrent episode of meningitis, it is not always possible to immediately be sure what the aetiology is. The initial assessment should include:

- An evaluation of the patient’s adherence to fluconazole consolidation and maintenance phase treatment (using self-reported and pharmacy refill data).
- An enquiry as to whether the patient has recently started ART to support a possible IRIS diagnosis.
- An LP to measure opening pressure, assess CSF inflammation and for a prolonged fungal culture (request 14 days of incubation in the laboratory) on at least 3 mL – 5 mL of CSF. There is no role for India ink staining or CSF/blood CrAg assays in establishing the cause of recurrence as these tests may remain positive for months to years even in patients after successful treatment – refer to Recommendation 2.
- If the CSF is culture-positive and non-adherence does not appear to be the cause, then fluconazole susceptibility testing should be considered. Susceptibility testing should be performed in an academic/reference laboratory. The panel recommends this when there has been at least one documented relapse despite reported good adherence (Recommendation 2).

If the cause of the recurrence is attributed to non-adherence, then the patient should be treated as for the first episode (Recommendation 3). The reasons for non-adherence should be explored and the patient should receive additional adherence counselling, preferably together with a treatment supporter. If the patient also interrupted ART, this should be re-initiated 4–6 weeks after induction antifungal treatment is re-started. Antiretroviral treatment may need to be adjusted if there is a concern that there has been virological failure.

Paradoxical cryptococcal IRIS occurs among patients treated for cryptococcal disease who start ART and develop a recurrence or worsening of clinical manifestations of cryptococcal disease. Immune reconstitution inflammatory syndrome is thought to be the result of an immunopathological reaction directed at residual fungal antigens at sites of disease.^40^ IRIS occurs on average 6 weeks after ART is commenced but delayed cases (> 1 year after ART initiation) have been described.^41^ Cryptococcal meningitis IRIS-associated mortality may be substantial.^42^ The most frequent manifestation is recurrence of symptoms of meningitis often with raised intracranial pressure. Typically, the CSF fungal culture is negative at the time of IRIS presentation – IRIS represents an immunological reaction rather than a microbiological recurrence. However, in cases where induction therapy was recent (< 2 months), the CSF fungal culture may still be positive. Other cryptococcal IRIS manifestations include lymphadenitis and cryptococcomas.^40^

In all patients with suspected paradoxical CM-IRIS, an LP should be performed to measure pressure and obtain a fungal culture incubated for up to 14 days. It is not possible to make a diagnosis of IRIS with certainty prior to excluding microbiological relapse on CSF fungal culture.

### Suspected cryptococcal meningitis-immune reconstitution inflammatory with mild symptoms

If the symptoms are mild, the panel recommends performing therapeutic LPs if there is raised intracranial pressure, providing analgesia and increasing the fluconazole dose to 1200 mg daily with regular review and follow-up of the CSF fungal culture result. If the CSF fungal culture is subsequently confirmed as negative, the dose of fluconazole can be reduced to what it was (800 mg or 200 mg daily depending on the timing of the CM-IRIS event).

### Suspected cryptococcal meningitis-immune reconstitution inflammatory syndrome with severe symptoms or clinical deterioration

If patients have severe symptoms or deteriorate with the approach above, the panel recommends treating with amphotericin B deoxycholate 1 mg/kg/day plus either flucytosine (100 mg/kg/day divided into four doses per day) or fluconazole 1200 mg daily until the CSF culture is confirmed as negative. If the CSF culture is still negative after 7 days’ incubation, amphotericin B can be stopped. If the fungal culture is positive by 7 days, then amphotericin B should be continued for 14 days if it is given with fluconazole (if it is given with flucytosine, then 7 days is sufficient). Daily therapeutic LPs may be required if opening pressure is raised. A CT brain scan should be considered as mass lesions and cerebral oedema can occur with IRIS. Analgesia should be provided. Among patients with severe IRIS who do not respond to the above treatment, corticosteroids should be considered: prednisone 1 mg/kg/day or an equivalent dose of dexamethasone for up to 2 weeks or until clinical improvement occurs, tapered over 2–6 weeks. Longer treatment may be required depending on the symptom response. The panel recommends that corticosteroids preferably should be used among patients with IRIS who are documented to be CSF fungal culture-negative and when other aetiologies (including tuberculous meningitis [TBM] and neurotropic viral infections) are excluded. However, if there is life-threatening neurological deterioration, corticosteroids should be started immediately.

Among patients with meningitis caused by fluconazole-resistant *Cryptococcus*, subsequent management should be discussed with a medical practitioner experienced in the treatment of CM. Such patients should receive induction therapy with amphotericin B again. Consolidation and maintenance options will depend on the fluconazole MIC and may include high doses of fluconazole, voriconazole or posaconazole with or without weekly amphotericin B infusions.^25^

Among patients with multiple relapses, it is important to document conversion of CSF from culture-positive to culture-negative before stopping amphotericin B and such cases should be discussed with a medical practitioner experienced in CM management and fluconazole susceptibility testing should be performed.

Distinguishing CM relapse or IRIS from TBM: Immune-suppressed HIV-seropositive adults are at high risk of disseminated TB, including TBM. Cerebrospinal fluid cell profiles and protein levels in CM are similar to those seen in TBM.^65^ However, a positive CrAg test or fungal culture is diagnostic for CM, and concomitant TBM is then less likely.^66^ All patients with CM relapse or cryptococcal IRIS should have diagnostic tests performed for concomitant TB with CSF and sputum GeneXpert Ultra assay, urinary lipoarabinomannan assay and chest radiograph (although pulmonary TB and cryptococcosis are difficult to distinguish radiologically).
